# The impact of COVID-19 on accelerating of immunosenescence and brain aging

**DOI:** 10.3389/fncel.2024.1471192

**Published:** 2024-12-10

**Authors:** Ludmila Müller, Svetlana Di Benedetto

**Affiliations:** Max Planck Institute for Human Development Center for Lifespan Psychology, Berlin, Germany

**Keywords:** COVID-19, long COVID, brain aging, neuroinflammation, immunosenescence, inflammaging, neurological disorders

## Abstract

The COVID-19 pandemic, caused by the novel coronavirus SARS-CoV-2, has profoundly impacted global health, affecting not only the immediate morbidity and mortality rates but also long-term health outcomes across various populations. Although the acute effects of COVID-19 on the respiratory system have initially been the primary focus, it is increasingly evident that the virus can have significant impacts on multiple physiological systems, including the nervous and immune systems. The pandemic has highlighted the complex interplay between viral infection, immune aging, and brain health, that can potentially accelerate neuroimmune aging and contribute to the persistence of long COVID conditions. By inducing chronic inflammation, immunosenescence, and neuroinflammation, COVID-19 may exacerbate the processes of neuroimmune aging, leading to increased risks of cognitive decline, neurodegenerative diseases, and impaired immune function. Key factors include chronic immune dysregulation, oxidative stress, neuroinflammation, and the disruption of cellular processes. These overlapping mechanisms between aging and COVID-19 illustrate how the virus can induce and accelerate aging-related processes, leading to an increased risk of neurodegenerative diseases and other age-related conditions. This mini-review examines key features and possible mechanisms of COVID-19-induced neuroimmune aging that may contribute to the persistence and severity of long COVID. Understanding these interactions is crucial for developing effective interventions. Anti-inflammatory therapies, neuroprotective agents, immunomodulatory treatments, and lifestyle interventions all hold potential for mitigating the long-term effects of the virus. By addressing these challenges, we can improve health outcomes and quality of life for millions affected by the pandemic.

## 1 Introduction

The unprecedented COVID-19 pandemic claimed millions of lives in a short period, and its threat continues to affect survivors through post-COVID syndrome, commonly known as long COVID (Davis et al., 2023). This condition is characterized by a diverse set of symptoms that persist long after the initial infection has resolved and can affect virtually every organ and system in the body. This multisystemic nature necessitates a multidisciplinary approach to diagnosis, treatment, and investigation. In addition to uncertainties about its pathogenesis, significant questions remain regarding the long-term trajectory of post-COVID conditions. Long COVID may present a potential additional public health crisis, with estimates suggesting that 1 in 5 COVID-19 survivors exhibit clinical manifestations consistent with this syndrome. The incidence of long COVID is valued at 10–30% in non-hospitalized cases, 50–70% in hospitalized cases, and 10–12% in vaccinated individuals, highlighting its widespread impact (Ceban et al., 2022; Davis et al., 2023).

A meta-analysis of 41 studies has identified several risk factors for developing long COVID, including female sex, older age, higher BMI, and smoking (Tsampasian et al., 2023). Women, especially those under 50, may be up to three times more likely to be diagnosed with long COVID. This disparity can be attributed to biological differences in the expression of angiotensin-converting enzyme-2 (ACE2) and transmembrane serine protease 2 (TMPRSS2) between genders, as well as immunological variations between sexes (Fernandez-de-Las-Penas et al., 2022; Klein and Flanagan, 2016; Tsampasian et al., 2023) highlighting the significance of the immunological component in the long COVID conditions.

In general, long COVID affects individuals across all age groups, with the highest diagnosis rates occurring between ages 36 and 50. As the majority of overall COVID-19 cases comprise non-hospitalized patients with mild acute illness, most cases of long COVID in absolute numbers are found in this group. Alarmingly, a significant proportion of these individuals develop debilitating symptoms, including severe fatigue, neurological conditions, respiratory issues, and cognitive impairments. These persistent symptoms can severely impact the quality of life, hindering individuals’ ability to return to work or perform daily activities.

Cognitive decline often associated with long COVID appears to affect all age groups, but older adults are particularly vulnerable to long-term cognitive impairments due to age-related neurodegenerative changes and immunosenescence (Arbula et al., 2024; Di Benedetto et al., 2017; Serrano Del Pueblo et al., 2024). In older individuals, the combination of chronic inflammation and neuroimmune dysregulation due to COVID-19 may accelerate processes linked to neurodegenerative diseases, resulting in more pronounced cognitive deficits, including memory loss, executive dysfunction, and impaired attention (Arbula et al., 2024; Ariza et al., 2023). However, studies have also shown cognitive changes in younger populations affected by long COVID (Tayeri et al., 2022). While typically less severe than in older adults, younger individuals may experience difficulties with attention, processing speed, and working memory—key functions that can impact daily activities and overall quality of life. Although these effects are often milder, the persistence of symptoms suggests that long COVID can lead to subtle but lasting changes in cognitive abilities across age groups.

The broad spectrum of long COVID manifestations places a significant burden on healthcare systems, which must adapt to manage and treat this emerging public health crisis effectively (Davis et al., 2023; Tsampasian et al., 2023). The widespread impact of COVID-19 across various age groups, particularly young and middle-aged individuals, and its long-term complications resembling those typically seen in the elderly (such as cardiovascular complications, diabetes, joint bone problems, depression, cognitive and neurodegenerative disorders) suggest that age-related conditions may appear at younger ages in the future (Tayeri et al., 2022).

The immune system plays a crucial role in the pathophysiology of COVID-19 and the subsequent development of post-COVID syndrome (Altmann et al., 2023; Files et al., 2021; Müller and Di Benedetto, 2023b). During the acute phase of infection, the immune response orchestrates the defense mechanisms to eliminate the virus, but an overactive or misdirected immune response can lead to tissue damage and inflammation, contributing to severe disease and long-term symptoms (Akbar and Gilroy, 2020; Müller and Di Benedetto, 2021).

Chronic inflammation, a key factor in the development and persistence of long COVID, can vary significantly among individuals due to genetic and environmental influences (Nasef et al., 2017). Current literature supports the hypothesis that host genetic factors influence susceptibility to COVID-19. Genetic factors, such as polymorphisms in genes encoding cytokines (e.g., IL-6, TNF-α) and immune-regulatory molecules, can modulate the inflammatory response (Choudhri et al., 2023; Nasef et al., 2017), making certain individuals more prone to heightened inflammation and prolonged immune dysregulation. Variants in genes related to the ACE2 receptor—the primary entry point for SARS-CoV-2—may also influence susceptibility to infection and subsequent inflammatory responses (Ren et al., 2022). Additional research is needed to fully identify relevant genetic variants and uncover the causal mechanisms behind these associations (Choudhri et al., 2023). Environmental factors—such as diet, physical activity, chronic stress, and exposure to pollutants—further influence baseline inflammation levels and immune response variability (Zhang et al., 2023). These environmental and genetic contributors may underlie disparities in long COVID outcomes across populations.

The immune system is in constant communication with neurological processes through a network of signaling molecules and pathways. This ongoing cross-talk is essential for maintaining homeostasis but can also contribute to pathological disease states (Bohmwald et al., 2024; Davis et al., 2023; Di Benedetto et al., 2017; Lord et al., 2024; Tsampasian et al., 2023). The complex interplay between the immune and neurological systems in long COVID involves a dynamic and often dysregulated interaction that can lead to prolonged symptoms affecting various body systems. Similarly, this intricate relationship is observed in the aging process, where immune system changes can influence neurological health and vice versa, highlighting the importance of these interconnected systems in both disease and aging contexts (Akbar and Gilroy, 2020; Müller and Di Benedetto, 2023b; Shafqat et al., 2024).

Moreover, the chronic inflammation and immune dysregulation associated with long COVID may accelerate neuroimmune aging in affected individuals (Lord et al., 2024; Shafqat et al., 2024). Neuroimmune aging refers to the progressive decline in the interactions between the nervous and immune systems as part of the aging process. It involves increased chronic inflammation (inflammaging), immune system dysfunction (immunosenescence), and neuroinflammation, which together contribute to cognitive decline, reduced neural plasticity, and heightened vulnerability to neurodegenerative diseases. This term highlights the interconnected deterioration of both the immune and nervous systems as organisms age. Such interrelated decline contributes to increased susceptibility to infections, neurodegenerative diseases, and overall morbidity and mortality (Dantzer, 2018; Davis et al., 2023; Hayley and Sun, 2021; Müller and Di Benedetto, 2023a). An increasing number of COVID-19 survivors experience cognitive and neurological impairment, with strong evidence pointing to a trajectory of accelerated aging and neurodegeneration. This potential change in the trajectory of aging could lead to an earlier onset of age-related diseases and conditions, such as neurodegenerative disorders, cardiovascular diseases, and metabolic syndromes, in this population (Davis et al., 2023; Foster et al., 2022; Müller and Di Benedetto, 2023b; Shafqat et al., 2024).

In the sections that follow we aim to explore the impact of COVID-19 on neuroimmune function and examine the potential mechanisms through which COVID-19 may accelerate neuroimmune aging – an area of growing concern given the long-term health implications for those infected with the virus. This research is crucial not only for addressing the current needs of long COVID patients but also for understanding how COVID-19 might alter the natural aging process, ultimately preparing healthcare systems to handle potential future pandemics with similar long-term sequelae.

## 2 Exploring the link between COVID-19 and immunosenescence

COVID-19 is associated with higher morbidity and mortality rates among older adults. A significant contributing factor is the aging immune system, which renders older individuals more susceptible to infections, less capable of effectively controlling them, and more prone to detrimental responses such as hyperinflammation and autoimmunity (Files et al., 2021; Müller and Di Benedetto, 2021). On the other hand, the infection itself may exacerbate features of immunosenescence, leading to accelerated immune aging. This phenomenon could affect not only older individuals but also those who experienced COVID-19 at a younger age, potentially changing the trajectory of their immune aging (Akbar and Gilroy, 2020; Altmann et al., 2023; Koff and Williams, 2020; Lucas et al., 2020).

**Figure 1:**
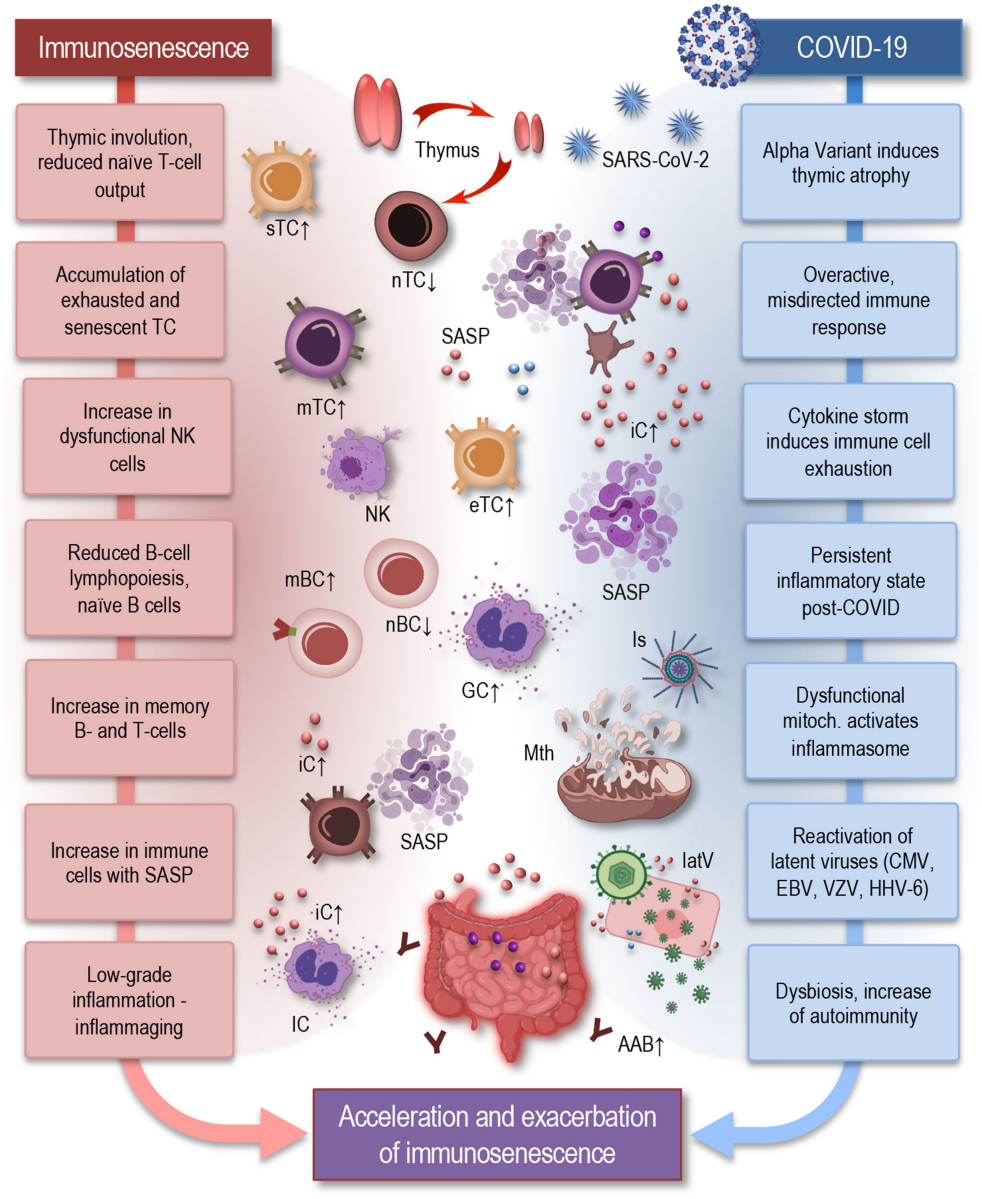
Parallels between immunosenescence and impact of COVID-19: acceleration and exacerbation of immune aging in long COVID. Hallmarks of immunosenescence (left) include thymic involution, which leads to diminished naïve T-cell output, accumulation of exhausted and senescent memory T cells, and an increase in NK cells with reduced cytotoxicity. Reduced B-cell lymphopoiesis leads to a decrease in naïve and regulatory B cells and an accumulation of memory B cells. Senescent T cells represent pro-inflammatory features, exhibiting a senescence-associated secretory phenotype (SASP) (Glass et al., 2010) immune cells also exhibit an inflammatory phenotype, producing pro-inflammatory cytokines even in the absence of infection. These changes contribute to inflammaging, which serves as a fundamental basis for numerous age-related diseases. COVID-19 (right) can exacerbate these features of immunosenescence by further inducing thymic atrophy, triggering an overactive and misdirected immune response, and causing reactivation of latent viruses. Dysfunctional mitochondria activate inflammasomes, contributing to a persistent inflammatory state. Additionally, gut dysbiosis, a hallmark of aging, may contribute to autoimmunity, neuroinflammation, dysautonomia, and metabolic disturbances—all signatures, seen also in long COVID. Together, these processes illustrate how COVID-19 can induce or accelerate the effects of immunosenescence, highlighting the complex interplay between chronic viral infections and the immune system aging. IC, immune cells; sTC, senescent T cell; nTC, naïve T cell; mTC, memory T cells; eTC, exhausted T cell; nBC, naïve B cell; mBC, memory B cell; SASP, senescence-associated secretory phenotype; NK, natural killer cell; iC, inflammatory cytokine; Is, inflammasome; Mth, mitochondria; latV, latent viruses; AAB, autoimmune antibody, EBV, Epstein–Barr virus; VZV, varicella-zoster virus; HHV-6, human herpesvirus 6; CMV, cytomegalovirus.

The characteristic feature of immunosenescence—the gradual decline of immune function with age—is the substantial remodeling of the immune system (Figure 1), leading to increased susceptibility to infections, reduced responses to vaccinations, and elevated risk of autoimmunity. Hallmarks of immunosenescence include thymic atrophy, which leads to diminished naïve T-cell output, accumulation of exhausted and senescent memory T cells, and an increase in natural killer (NK) cells with reduced cytotoxicity (Aiello et al., 2019; Fulop et al., 2021; Hazeldine et al., 2012; Liu et al., 2023; Müller et al., 2019; Pawelec, 2018). Furthermore, advancing age is associated with reduced B-cell lymphopoiesis, leading to a decrease in naïve and regulatory B cells and an accumulation of memory B cells. Additionally, there is a shift toward Th17 polarization and an expansion of regulatory T cells (Tregs) with impaired suppressive capacity (Wu et al., 2021).

Immunosenescence affects both the innate and adaptive immune systems. In the innate immune system, aging macrophages exhibit reduced phagocytic capacity and heightened pro-inflammatory cytokine production. In the adaptive immune system, aging T cells display reduced proliferative potential and functional exhaustion, leading to impaired immune surveillance and response (Aiello et al., 2019; Alves and Bueno, 2019). The senescent T cells exhibit pro-inflammatory features, characterized by a senescence-associated secretory phenotype (SASP), which includes the secretion of various pro-inflammatory cytokines, such as IL-6, IL-1β, and TNF-α, as well as chemokines like CXCL8 and CCL2. This SASP not only contributes to the chronic, low-grade inflammation observed in aging but may also alter the local tissue environment, promoting immune dysfunction, tissue damage, and reduced immune surveillance. Macrophages in older adults also exhibit an inflammatory phenotype, producing pro-inflammatory cytokines such as IL-6, TNF-α, and IL-1β even in the absence of infection. These changes contribute to a pro-inflammatory state in older adults, termed inflammaging, which serves as a fundamental basis for numerous age-related diseases, including neurodegenerative disorders and chronic inflammatory conditions (Cunha et al., 2020; Franceschi et al., 2017; Fulop et al., 2021; Gruver et al., 2007; Liu et al., 2023; Müller et al., 2013). During COVID-19, this pre-existing immune dysfunction is exacerbated, with studies showing that aged T cells and macrophages have an amplified inflammatory profile in response to SARS-CoV-2, driving severe inflammation and poor outcomes in elderly patients (Omarjee et al., 2020; Pietrobon et al., 2020). Understanding how these immune components are affected by both aging and COVID-19 is crucial for addressing the long-term impact of the virus on older populations.

COVID-19 may exacerbate immunosenescence through several mechanisms, which can be both acute and persistent. In patients with severe COVID-19, a cytokine storm is often triggered, characterized by an excessive release of pro-inflammatory cytokines such as IL-6, TNF-α, and IL-1β. This uncontrolled inflammatory response is particularly detrimental in older adults, where immune senescence already contributes to a pro-inflammatory baseline (Alvarez-Heredia et al., 2023). Aging T cells, for example, exhibit markers of exhaustion, such as PD-1 and TIM-3, which limit their proliferative capacity and ability to mount an effective immune response (Wu et al., 2021). In addition, senescent immune cells, such as CD8^+^ T cells, show a senescence-associated secretory phenotype, releasing CXCL8, CCL2, and GM-CSF, further amplifying inflammation and contributing to tissue damage (Pius-Sadowska et al., 2022). The persistence of these senescent cells and their SASP may exacerbate the cytokine storm, leading to worse outcomes in aged populations. This specific immune dysregulation highlights the complex interplay between immune exhaustion, senescence, and cytokine overproduction, providing a clearer understanding of how aging contributes to severe COVID-19 pathology (Bauer and Fuente Mde, 2016; Domingues et al., 2020; Müller and Di Benedetto, 2021).

Thus, the virus-induced cytokine storm can cause immune cell exhaustion, while chronic inflammation can promote the accumulation of senescent immune cells (Shafqat et al., 2024; Tizazu et al., 2022). Together, these effects can lead to a more rapid decline in immune function and progression of immunosenescence (Figure 1). During the initial phase of infection, the immune system can be overwhelmed by the virus, leading to a heightened state of immune activation (Alvarez-Heredia et al., 2023; Müller and Di Benedetto, 2021; Wilk et al., 2020; Zheng et al., 2020). This acute stress can accelerate the aging of immune cells, causing them to exhibit characteristics typical of older immune systems, such as reduced proliferation capacity, diminished response to new infections, and increased production of pro-inflammatory cytokines. During the acute phase of COVID-19, research has demonstrated signs of both an exhausted and aged immune phenotype in patients. This includes CD8 T cells and NK cells exhibiting reduced production of interleukin (IL)-2 and interferon (IFN)-γ, diminished granzyme expression and degranulation, and an increased expression of the inhibitory receptor NKG2A (Wilk et al., 2020; Zheng et al., 2020). These alterations in immune function are more pronounced with increasing severity of the disease, suggesting a direct link between these immune characteristics and a compromised ability to respond to the infection (Lord et al., 2024).

Furthermore, the interplay between COVID-19 and immunosenescence not only affects immediate outcomes but may also have long-term implications for older and also mid-aged adults. Post-infection, the long-term effects of the virus can continue to influence the immune system (Davis et al., 2023; Koff and Williams, 2020; Müller and Di Benedetto, 2023b; Tsampasian et al., 2023). In some COVID-19 patients, particularly those with severe disease, the immune system remains activated long after the acute infection has resolved (Lord et al., 2024; Müller and Di Benedetto, 2021). This chronic immune activation is associated with ongoing inflammation and further immune cell exhaustion. The persistent inflammatory state seen in COVID-19 survivors may therefore lead to sustained immune system dysfunction and contribute to accelerated immune aging. A similar state of accelerated immunosenescence has been observed in a younger cohort of traumatic injury patients, suggesting that an acute challenge can negatively influence immune aging (Foster et al., 2022).

Recent studies observe a decay in long COVID cases, likely due to the evolving nature of the virus variants (Bohmwald et al., 2024). Acute infection with the Alpha variant causes significant thymic atrophy, a key indicator of immunosenescence, leading to the depletion of naïve T cells (Goncalves et al., 2023). This characteristic is not observed with the Delta and Omicron variants, implying that individuals infected earlier in the pandemic may be more susceptible to developing immunosenescence (Shafqat et al., 2024). This finding highlights the variant-specific pathophysiology of long COVID and its broader implications for understanding the disease’s progression.

Using a composite score of immune aging known as IMM-AGE, researchers have demonstrated that SARS-CoV-2 infection accelerates immunosenescence (Lord et al., 2024). They supposed that antigenic stimulation during viral infection can lead to telomere shortening and the emergence of more highly differentiated EMRA T cells, as well as exhausted and senescent T cells. Studies conducted on individuals recovering from COVID-19, 3–5 and 8 months post-infection, have shown sustained high levels of IL-6 and two further anti-viral cytokines (IFN-β and IFN-λ1), which were associated with persistent symptoms of long COVID (Lord et al., 2024; Phetsouphanh et al., 2022). So-called footprint of long COVID was characterized by prolonged inflammation (lasting over 8 months) induced by specific innate immune cells, such as monocytes and plasmacytoid dendritic cells, along with the activation of CD8^+^ memory T-cell subsets expressing PD-1 (Altmann et al., 2023; Phetsouphanh et al., 2022).

Persistent inflammation, similar to the chronic inflammatory state observed in elderly individuals, is a characteristic feature in many patients with long COVID conditions. Due to the biological and pathophysiological heterogeneity of long COVID, inflammaging might be particularly evident in a subset of individuals with an inflammatory subtype. This subtype is marked by persistent elevations in IL-1, IL-6, TNFα, type I and II interferons, neutrophil activation, altered B-cell memory, and autoreactivity (Shafqat et al., 2024; Talla et al., 2023). Subtyping individuals with long COVID based on their immune cell phenotype and cytokine profile, researches identified a distinct group of patients with persistent inflammation. This group is characterized by heightened innate and adaptive immune activation, alongside a potential loss of immune-regulatory mediators and adaptive immune cells (Ruffieux et al., 2023)—features shared with immunosenescence. Moreover, persistent elevations of CD40 in critically ill COVID-19 patients, observed even 6 months post-infection, indicate sustained T-cell activation and chronic inflammation (Klein et al., 2023). Additionally, a significant area of overlap involves the reactivation of latent herpesviruses such as Epstein–Barr virus (EBV), varicella-zoster virus (VZV), human herpesvirus 6 (HHV-6), and cytomegalovirus (CMV). This reactivation is accompanied by increased adaptive immune reactivity, further complicating the clinical picture and potentially exacerbating the long-term effects of COVID-19 (Klein et al., 2023; Müller and Di Benedetto, 2024; Pius-Sadowska et al., 2022).

The NLRP3 inflammasome plays a central role in the immune response to viral infections, including SARS-CoV-2, by sensing damage-associated molecular patterns (DAMPs) and pathogen-associated molecular patterns (PAMPs). Upon activation, the NLRP3 inflammasome promotes the release of pro-inflammatory cytokines, such as IL-1β and IL-18, which are key mediators of the inflammatory response seen in severe COVID-19 cases (Vora et al., 2021). However, chronic NLRP3 activation is also implicated in immunosenescence and is associated with aging (Ferrucci and Fabbri, 2018; Liang et al., 2024). This intersection between NLRP3 activation, COVID-19, and aging is crucial for understanding how long COVID may exacerbate immune aging.

In the context of COVID-19, NLRP3 inflammasome activation can drive prolonged and excessive inflammation, contributing to cytokine storm in severe cases and potentially leading to long-term immune dysregulation (Liang et al., 2024; Pan et al., 2021; Vora et al., 2021). Persistent NLRP3 activation, particularly in elderly individuals or those with pre-existing conditions, may exacerbate chronic inflammation—a hallmark of both inflammaging and long COVID. Studies have shown that NLRP3 inflammasome activation is elevated in older adults, linking it directly to age-related diseases, including neurodegenerative disorders (Ferrucci and Fabbri, 2018; Heneka et al., 2020; Vora et al., 2021). This suggests that COVID-19-induced activation of the NLRP3 pathway could accelerate the aging process by amplifying inflammation. In COVID-19 patients, particularly those who experience severe infection or long COVID, the activation of the NLRP3 inflammasome may perpetuate this chronic inflammatory state, leading to immune exhaustion, neuroinflammation, and worsening of age-related conditions.

Inflammatory regulators like the NLRP3 inflammasome may be activated by dysfunctional mitochondria, thereby linking mitochondrial dysfunction to inflammaging and cellular senescence (Guo et al., 2023). Dysfunctional mitochondria may also be associated with other shared pathways of aging and COVID-19, including inflammation and IFN responses (He et al., 2023), cellular senescence (Miwa et al., 2022), endothelial dysfunction, coagulopathy (Costa et al., 2022; Molnar et al., 2024), and dysbiosis (Saleh et al., 2020; Shafqat et al., 2024). Thus, integrating NLRP3 inflammasome activation into the broader discussion of long COVID and immunosenescence, we can see how this pathway not only drives the acute inflammatory response but also contributes to the accelerated aging of the immune system.

Gut microbiome dysbiosis, a hallmark of aging, may contribute to autoimmunity, neuroinflammation, dysautonomia, and metabolic disturbances – all seen in long COVID (Altmann et al., 2023; Ghosh et al., 2020; Shafqat et al., 2024). Indeed, dysbiosis, characterized by reduced beneficial taxa and decreased diversity, is also a key feature of long COVID (Al-Aly and Topol, 2024; Altmann et al., 2023). COVID-19 patients often show significant alterations in the composition of gut microbiota, particularly affecting bacterial phyla such as *Firmicutes, Bacteroidetes, Actinobacteria*, and *Proteobacteria*. Research suggests a reduction in beneficial *Firmicutes* and *Bacteroidetes* and an increase in potentially harmful *Proteobacteria*, correlating with increased gut permeability and systemic inflammation. Additionally, decreases in beneficial *Bifidobacterium* populations are associated with an increased risk of invasion by potentially harmful opportunistic pathogens (Fakharian et al., 2023). These microbiota shifts can promote a pro-inflammatory state, driving immune dysregulation and neuroinflammation, which are key components of long COVID (Raj et al., 2024; Su et al., 2024; Zuo et al., 2020).

Furthermore, reduced levels of short-chain fatty acids (SCFAs)—beneficial metabolites produced by gut bacteria—have been observed in long COVID patients. SCFAs play a crucial role in maintaining gut and immune health by modulating inflammation and supporting regulatory T-cell function (Mann et al., 2024; Raj et al., 2024). The depletion of these metabolites may exacerbate systemic inflammation and impair the immune response, contributing to persistent symptoms like fatigue, cognitive decline, and gastrointestinal issues in long COVID patients (Wlodarczyk et al., 2022; Zhang et al., 2022).

Gut microbiome dysbiosis has been shown to correlate with symptoms such as insomnia, poor memory, anxiety, and respiratory issues (Liu et al., 2022; Silva et al., 2020; Vestad et al., 2022). Recent studies link distinct microbiome profiles to specific long COVID endotypes (Shafqat et al., 2024; Su et al., 2024). To better understand the pathophysiological mechanisms of long COVID, it is crucial to consider the significant role of the microbiome in regulating neuroimmune interactions through the “gut-brain axis” (Flux and Lowry, 2020; Valles-Colomer et al., 2019; Yang et al., 2020).

The presence of autoantibodies (AABs) is another significant factor in both immunosenescence and long COVID. In COVID-19 patients, the immune system may produce AABs that target self-antigens, contributing to autoimmune-like symptoms (Akbari et al., 2023). This dysregulated immune response can persist long after the acute phase of infection, as seen in patients with long COVID. Many studies investigating autoimmunity following SARS-CoV-2 infection have highlighted the induction of a wide range of autoantibodies (Richter et al., 2021; Sacchi et al., 2021; Wallukat et al., 2021). Early investigations into immune responses during acute COVID-19 revealed that individuals with pre-existing anti-type I interferon AABs tended to experience more severe infection (Akbari et al., 2023). Additionally, SARS-CoV-2 appears capable of triggering the production of a diverse array of new AABs (Altmann et al., 2023; Müller and Di Benedetto, 2023b; Wallukat et al., 2021; Wang et al., 2021). Research on autoantibody levels in serum has revealed a high prevalence of antibodies targeting skin, skeletal muscle, and cardiac tissue (Akbari et al., 2023; Chang et al., 2021; Müller and Di Benedetto, 2023b; Richter et al., 2021). These AABs may exacerbate inflammatory processes, particularly in individuals with pre-existing immunosenescence, where the immune system’s ability to distinguish self from non-self becomes impaired. The persistent activation of autoimmunity via AABs, combined with the dysregulated cytokine release and inflammatory state described earlier, likely plays a key role in prolonging the immune dysfunction and multisystem symptoms seen in long COVID (Akbari et al., 2023).

Taken together, both aging and COVID-19 are associated with an increased inflammatory state and immune dysregulation, which can lead to similar pathological outcomes. The virus accelerates aging by exacerbating processes like inflammation, mitochondrial dysfunction, and cellular senescence, all of which show striking parallels with age-related declines in immune function and physiological resilience. Thus, the chronic inflammation and immune dysregulation associated with long COVID may induce and accelerate immune aging through different mechanisms, mirroring the immune senescence seen in the elderly. This has profound implications for the long-term health of COVID-19 survivors. In the following section, we will explore how immune dysregulation induced by both aging and COVID-19 can exacerbate sustained neuroinflammatory changes in the brain, leading to the disruption of normal brain function and potentially resulting in significant long-term consequences.

## 3 Neuroimmune interactions: the dual impact of aging and COVID-19

**Figure 2:**
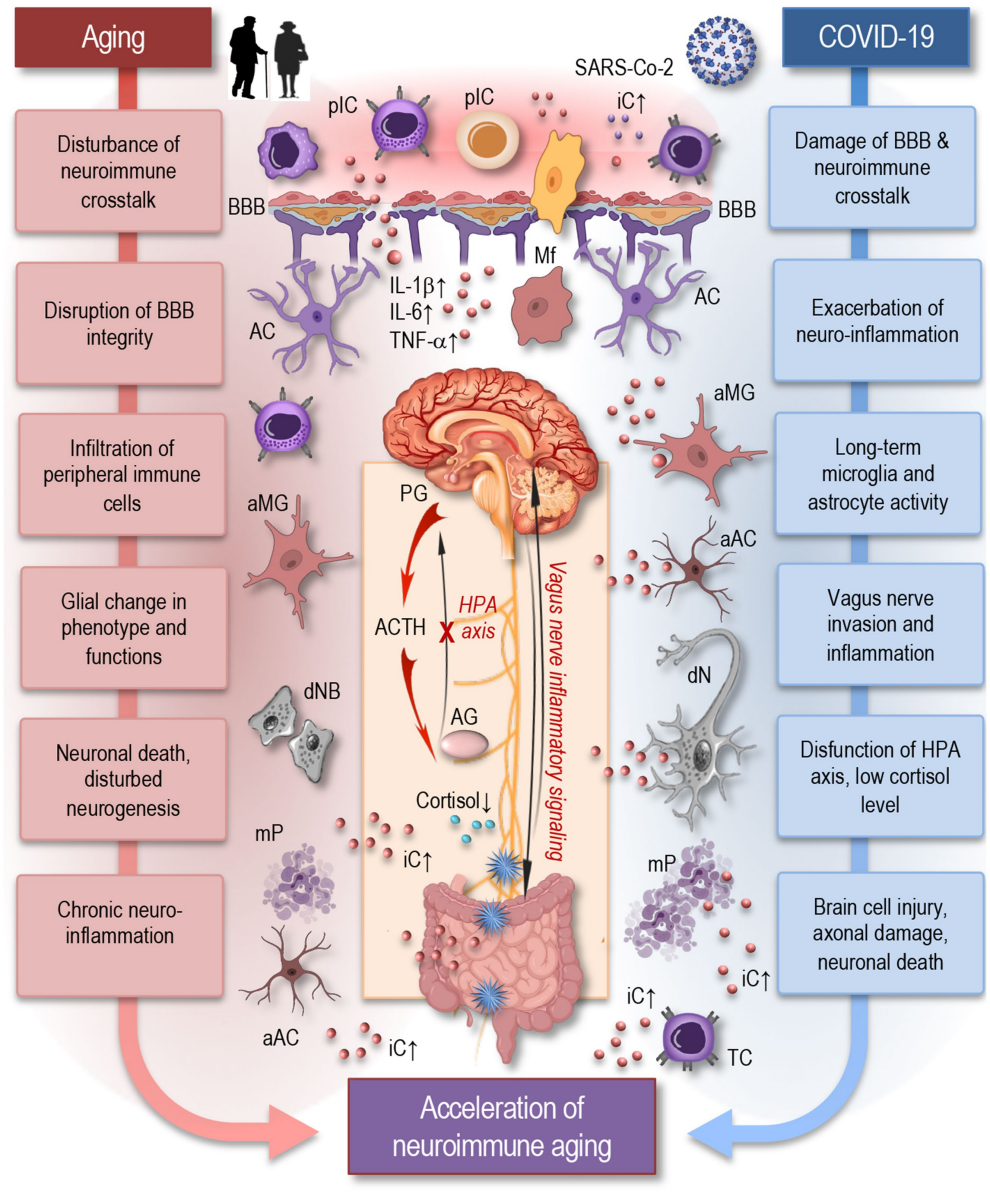
Aging and COVID-19 share significant overlaps in their impact on neuroimmune interactions and may accelerate neuroimmune aging. As depicted in this simplified figure, both aging and COVID-19 can induce neuroinflammation through the accumulation of senescent cells, persistent microglia and astrocytes’ activation, and increased pro-inflammatory cytokine production, such as IL-1β, IL-6, and TNF-α. Similar to aging, SARS-CoV-2 infection can cause significant damage to the blood–brain barrier, facilitating the infiltration of peripheral immune cells and inflammatory mediators into the brain, which disrupts neuroimmune crosstalk and accelerates neuroimmune aging. Inflammatory signaling via the vagus nerve exacerbates neuroinflammatory responses, potentially influencing systemic inflammation and immune responses. The HPA axis experiences dysfunction, resulting in decreased cortisol levels, further exacerbating neuroinflammation. These factors create a chronic inflammatory environment that can damage neurons, disrupt synaptic connectivity, and impair neuroimmune interactions leading to accelerated neuroimmune aging. pIC, peripheral immune cells; iC, inflammatory cytokines; BBB, blood-brane-barrier; Mf, macrophage; MG, microglia; AC, astrocyte; aMG, activated microglia; aAC, activated astrocyte; IL, interleukin; TNF, tumor necrosis factor; PG, pituitary gland; ACTH, adrenocorticotropic hormone; AG, adrenal gland; HPA, the hypothalamic–pituitary–adrenal axis; dNB, damaged neuroblast; dN, damaged neuron; mP, misfolded proteins; TC, T cell.

The nervous and immune systems are interconnected through various pathways, including neural, humoral, and cellular routes (Figure 2). Neuroimmune crosstalk is essential for maintaining homeostasis and responding to stressors. For example, the vagus nerve can modulate immune responses by transmitting signals from the brain to the gut and other organs (Dantzer, 2018; Di Benedetto et al., 2017; Matejuk et al., 2021). Similarly, immune cells can produce cytokines and other immune mediators that affect brain function (Boahen et al., 2023; Dantzer, 2018). Furthermore, immune cells work with the neurovascular unit to preserve the integrity of the blood–brain barrier (BBB), ensuring selective permeability and protecting the central nervous system (CNS) from harmful substances in the bloodstream.

The neuroimmune system encompasses a complex network of neural and immune cells that interact to maintain homeostasis in the CNS and respond to injury or infection (Dantzer, 2018; Hayley, 2014). Key components of this system include astrocytes, microglia, oligodendrocytes, and neurons—all of which play critical roles in CNS immune responses (Boahen et al., 2023; Matejuk et al., 2021). Astrocytes, the most abundant glial cells, regulate the BBB and modulate inflammatory responses in the CNS. They may become reactive during viral infection, contribute to neuroinflammation and release cytokines that may influence both neuronal and immune cell activity. Microglia, the resident immune cells of the brain, act as the CNS’s first line of defense. Upon inflammatory conditions, microglia may become activated, releasing pro-inflammatory cytokines and further driving neuroinflammatory processes. Oligodendrocytes are responsible for producing myelin, which insulates axons and ensures efficient signal transmission between neurons. Inflammatory conditions may impair myelination, contributing to cognitive and motor dysfunction (Hayley, 2014; Matejuk et al., 2021). Neurons themselves are susceptible to damage from prolonged neuroinflammation, which can lead to impaired neural communication, and ultimately neuronal death.

Aging is known to disrupt neuroimmune crosstalk, leading to increased vulnerability to infections and neurodegenerative diseases. Age-related decline in immune function within the brain can result in chronic neuroinflammation, synaptic dysfunction, and neuronal damage, disturbed neurogenesis, which are characteristic features of many neurological disorders (Glass et al., 2010; Wendimu and Hooks, 2022). The aging brain shows increased levels of pro-inflammatory cytokines, which can affect neural function and plasticity. An excess of soluble inflammatory mediators in the blood circulation, including cytokines (e.g., TNF-*α*, IL-1β, IL-6, IFN-γ), pathogen-associated molecules (e.g., LPS, viral nucleic acids), complement components, sphingosine, prostaglandins, and kinins, can adversely affect the BBB. Persistent exposure to these mediators disrupts endothelial barriers, allowing immune cells and inflammatory cytokines to penetrate the brain parenchyma, creating an inflammatory environment. This activates microglia and other resident cells like astrocytes, neurons, and oligodendrocytes, as well as infiltrating immune cells such as monocytes, T cells, and macrophages (Figure 2). The resulting neuroinflammation can cause structural damage, impair regeneration, induce neuronal cell death, and negatively impact brain function by modulating synaptic remodeling (Colonna and Butovsky, 2017; Di Benedetto et al., 2017; Dulken et al., 2019; Gemechu and Bentivoglio, 2012; Groh et al., 2021; Jin et al., 2021; Mrdjen et al., 2018).

Immune dysregulation from COVID-19 can exacerbate sustained neuroinflammatory changes in the brain (Figure 2), leading to long-term damage and disruption of normal brain function (Hayley and Sun, 2021; Radke et al., 2024; Shafqat et al., 2024). Markers of brain injury, including neurofilament light chain (NFL), indicative of axonal damage, and glial fibrillary acidic protein (GFAP), a marker of astrocyte reactivity, were shown to be elevated in serum of COVID-19 patients. These elevations correlated with disease severity and increased levels of inflammatory cytokines such as IL-1β, TNFα, and IL-6 (Needham et al., 2022; Shafqat et al., 2024). While circulating pro-inflammatory cytokine levels were shown to normalize in recovering patients, especially those with mild to moderate infections, NFL and GFAP remained elevated, indicating ongoing brain injury even after acute disease resolution (Michael et al., 2023; Shafqat et al., 2024). Microglial activation was demonstrated to persist up to 7 weeks after clearing a mild COVID-19 respiratory infection (Fernández-Castañeda et al., 2022). Total tau protein, another brain injury marker, was exclusively raised in serum of patients with long COVID-associated neurological complications, but not in those without long COVID (Shafqat et al., 2024). Specific anatomical regions were found to be more affected in long COVID patients, with NFL, GFAP, and total-tau elevated in those who developed persistent cognitive deficits that did not fully recover at one-year follow-up. Reduced cortical thickness in the superior temporal gyrus was associated with NFL elevations (Shafqat et al., 2024).

In the context of aging, NFL levels in serum and cerebrospinal fluid (CSF) have been shown to increase in neurologically normal individuals as well as those with neurodegenerative diseases (Bacioglu et al., 2016). Serum NFL levels were found to increase nonlinearly after age 60, indicating accelerated neuronal injury at advanced ages, with premature increases associated with accelerated brain volume loss (Kalil et al., 2021; Shafqat et al., 2024). Additionally, plasma GFAP and NFL levels have been shown to robustly predict long-term dementia risk in cognitively healthy adults in the UK Biobank (Guo et al., 2024). These findings strongly suggest that some COVID-19 patients develop an accelerated brain aging phenotype (Guo et al., 2022). Indeed, compared to 2,974 matched controls, those with long COVID-related neurological complications at 1-year post-hospital admission were found to exhibit brain changes equivalent to aging from 50 to 70 years old (Shafqat et al., 2024).

Research has demonstrated that neuroinflammation can lead to dysregulation of glial and neuronal cells, resulting in neural circuit dysfunction, which adversely affects cognitive and neuropsychiatric functions (Colonna and Butovsky, 2017; Monje and Iwasaki, 2022). COVID-19 may accelerate neuroimmune aging by exacerbating these age-related changes. The virus-induced cytokine storm can lead to a sustained pro-inflammatory state, both systemically and within the CNS. This persistent inflammation can impair neural function and promote neurodegenerative processes (Domingues et al., 2020; Matejuk et al., 2021; Müller and Di Benedetto, 2023a). Peripheral immune cells, such as T cells and monocytes, can infiltrate the CNS during severe inflammation or BBB disruption, exacerbating neuroinflammatory conditions. The interaction between these peripheral immune cells and CNS-resident glial cells is crucial in shaping the neuroimmune response to SARS-CoV-2, potentially leading to prolonged neurological consequences in long COVID patients (Needham et al., 2022; Raony et al., 2020; Tang et al., 2022; Walitt and Johnson, 2022).

Additionally, chronic immune activation and immunosenescence induced by COVID-19 can further disrupt neuroimmune crosstalk, leading to a more rapid decline in both nervous and immune system function (Hayley and Sun, 2021; Matejuk et al., 2021; Müller and Di Benedetto, 2023a). Concurrently, the aged immune system is less efficient at mounting effective responses to pathogens and resolving inflammation. These changes may create a vicious cycle of chronic inflammation and neurodegeneration (Dantzer, 2018; Di Benedetto et al., 2017; Hayley, 2014; Matejuk et al., 2021).

Upregulated type I IFN responses may be a shared characteristic between aging and long COVID. For example, COVID-19 appears to induce inflammation in the choroid plexus, similar to the type I IFN-related inflammation observed with aging. This inflammation sends chemokine-rich pro-inflammatory signals to brain parenchymal glial cells and cortical neurons. Despite its importance in anti-SARS-CoV-2 immunity, the type I IFN response may contribute to neuroinflammation in the choroid plexus, paralleling aging-related changes, due to the high ACE2 expression on choroid epithelial cells (Shafqat et al., 2024; Suzzi et al., 2023).

It was suggested that SARS-CoV-2 has the potential to directly invade the vagus nerve, initiating inflammatory changes that may lead to its dysfunction (Figure 2). These inflammatory signals can then travel retrograde along the nerve, reaching the brain and causing neuroinflammation (Matejuk et al., 2021; Müller and Di Benedetto, 2023a). This pathway not only disrupts autonomic functions regulated by the vagus nerve but also contributes to the broader neuroinflammatory processes observed in COVID-19, potentially exacerbating neurological symptoms and cognitive impairments associated with long COVID (Woo et al., 2023).

In addition to its well-known anti-inflammatory effects via efferent pathways, the vagus nerve also plays a critical role in transmitting inflammatory signals from the periphery to the CNS through afferent pathways. Vagal afferent fibers are responsible for sensing peripheral inflammation and communicating these signals to the brain, where they can modulate CNS responses, particularly through regions such as the hypothalamus and brainstem (Caravaca et al., 2022; Jin et al., 2024; Radke et al., 2024). This process is crucial in the context of COVID-19, as persistent inflammation in peripheral organs—such as the lungs or gastrointestinal tract—may be transmitted to the CNS via the vagal afferents, contributing to neuroinflammation and neurological symptoms like brain fog, cognitive dysfunction, and fatigue (Radke et al., 2024).

Indeed, the vagus nerve’s role in gut-brain interactions is particularly relevant in long COVID, which is frequently associated with gut dysbiosis. Gut dysbiosis, characterized by imbalances in the gut microbiota, can induce inflammatory responses in the gastrointestinal tract (Aktas and Aslim, 2020; Raj et al., 2024; Zuo et al., 2020). The vagus nerve, through its afferent fibers, may sense these inflammatory changes and relay them to the CNS, thereby contributing to the neuroinflammatory processes observed in long COVID patients. This bidirectional communication between the gut and brain, often referred to as the gut-brain axis, plays a crucial role in maintaining neuroimmune homeostasis. Disruptions in this axis due to gut dysbiosis and inflammation in long COVID may exacerbate neurological symptoms, further highlighting the importance of the vagus nerve in modulating neuroimmune interactions (Finsterer, 2024; Jin et al., 2024; Lladós et al., 2024).

Another route the virus may use to access the CNS is through the olfactory bulb. SARS-CoV-2 has been shown to invade the CNS via this olfactory pathway, a well-established entry point for viruses, potentially contributing to neuroinflammatory processes and the neurological complications observed in long COVID (Wellford and Moseman, 2024). The olfactory pathway may act in concert with the vagus nerve to amplify CNS inflammation, creating a more comprehensive understanding of how SARS-CoV-2 can affect the brain and contribute to long-term neurological symptoms.

The hypothalamic–pituitary–adrenal (HPA) axis plays a critical role in regulating the immune system, particularly through the release of cortisol, which has potent anti-inflammatory effects. However, as individuals age, HPA axis dysregulation becomes more common, leading to altered cortisol rhythms, blunted stress responses, and diminished control over inflammatory processes. This dysregulation is a key contributor to immunosenescence and is closely linked to inflammaging (Bellavance and Rivest, 2014; Gardner et al., 2019; Raony et al., 2020; Spindler et al., 2023). In the context of long COVID, HPA axis dysfunction may exacerbate the already declining immune function seen in aging individuals. The chronic inflammation that persists in long COVID patients may be partly driven by an inability of the HPA axis to properly regulate the immune system’s inflammatory response (Jensterle et al., 2022).

Altered intercellular communication in long COVID often presents as low serum cortisol levels. This phenomenon is one of the most consistently observed and significant factors linked to long COVID and may partly explain the persistent fatigue and immune dysregulation seen in affected individuals (Shafqat et al., 2024; Woo et al., 2023). Low cortisol production by the adrenal gland is typically balanced by an increase in adrenocorticotropic hormone (ACTH) release by the pituitary gland. However, this compensation did not occur, indicating dysfunction in the HPA axis and may also suggest an underlying neuroinflammatory process. Low cortisol levels have also been observed in individuals with myalgic encephalomyelitis/chronic fatigue syndrome (ME/CFS), highlighting a similar endocrine pattern. This similarity suggests that both long COVID and ME/CFS may share common pathways of neuroimmune dysregulation (Davis et al., 2023; Klein et al., 2023).

The intersection of HPA axis dysregulation and immune aging is particularly important in understanding the long-term consequences of COVID-19 in older adults (Jensterle et al., 2022). The persistent stress on the immune system caused by both the aging process and COVID-19 may accelerate immunosenescence, further weakening immune surveillance and increasing susceptibility to infections, autoimmunity, and chronic inflammatory diseases. This dual burden of immune aging and COVID-19 could also contribute to the progression of neurodegenerative conditions, as the brain remains more vulnerable to inflammatory damage when the HPA axis is unable to control inflammatory responses effectively (Bellavance and Rivest, 2014; Gardner et al., 2019; Jensterle et al., 2022). By impairing the body’s ability to effectively regulate immune responses, HPA axis dysfunction may amplify the long-term health consequences of COVID-19, particularly in aging populations.

In general, chronic neuroinflammation and immune activation are key drivers of neurodegenerative diseases such as Alzheimer’s disease, Parkinson’s disease, and multiple sclerosis. COVID-19 may increase the risk of developing these conditions or accelerate their progression in individuals who are already affected. The persistent inflammatory state induced by the virus can promote the aggregation of misfolded proteins, neuronal death, and other pathological processes associated with neurodegeneration (Domingues et al., 2020; Heneka et al., 2020; Leite et al., 2021).

Further of concerning potential long-term consequences of COVID-19 is cognitive decline. Persistent neuroinflammation and BBB disruption can impair synaptic function and plasticity, leading to deficits in learning and memory. Studies have reported cognitive impairments in COVID-19 survivors, even in those with mild to moderate disease (Heneka et al., 2020; Suzzi et al., 2023; Zhao et al., 2024). These impairments may persist or worsen over time, particularly in older adults who are already at risk for cognitive decline, but they can also affect middle-aged and even young individuals.

Long COVID has been associated with declines in multiple cognitive functions, including memory, executive function, and working memory (Al-Aly and Rosen, 2024; Arbula et al., 2024; Ariza et al., 2023; Pallanti et al., 2023; Serrano Del Pueblo et al., 2024). Studies have documented impairments in episodic memory, where 48% of patients with long COVID struggle to recall recent events. It was observed that 27% of patients also showed impairments in overall cognitive function, particularly in attention, working memory, processing speed, and verbal fluency (Serrano Del Pueblo et al., 2024). Recent findings revealed attention and memory difficulties (Arbula et al., 2024) and deficits in executive functions (Ariza et al., 2023; Pallanti et al., 2023) in post-COVID patients, which affect planning, decision-making, and problem-solving abilities. These findings underscore the broad and lasting cognitive impacts of long COVID across various functional domains, impacting daily tasks and overall quality of life.

Thus, COVID-19 may stimulate and accelerate neuroimmune aging by exacerbating the age-related changes, such as chronic inflammation, immune dysregulation, and neuroinflammation. This parallel suggests that COVID-19 can act as an accelerant, intensifying the neuroinflammatory processes typically associated with aging and potentially hastening the onset of neurodegenerative conditions, such as Alzheimer’s and Parkinson’s diseases, as well as cognitive decline.

## 4 Insights into potential interventions and therapeutic strategies

In the previous sections we have seen that long COVID might not only mimic but also exacerbate the natural aging process in both immune and neurological systems, potentially accelerating the onset and progression of age-related diseases. Understanding these overlaps can provide valuable insights into both conditions, offering new avenues for therapeutic interventions and management strategies.

Given the crucial role of inflammation in COVID-19-related neuroimmune aging, anti-inflammatory therapies may offer potential benefits. Nonsteroidal anti-inflammatory drugs (NSAIDs) and corticosteroids are examples of treatments that can reduce inflammation. Immunomodulatory therapies that can restore immune function and reduce chronic inflammation may help mitigate the effects of COVID-19 on immune aging (Shafqat et al., 2024; Tay et al., 2020; Wyss-Coray, 2016). Examples of such therapies include cytokine inhibitors, immune checkpoint inhibitors, and senolytics, which selectively eliminate senescent cells. These therapies have the potential to improve immune function and reduce the risk of age-related diseases (Kirkland and Tchkonia, 2020; Müller and Di Benedetto, 2023a; Shafqat et al., 2024; Stahl and Brown, 2015). However, the timing and dosage of these therapies need to be carefully managed to avoid suppressing the necessary immune response to the virus.

In managing long COVID, immunomodulatory therapies must be carefully considered alongside preexisting treatments for conditions like diabetes and cardiovascular disease, as these comorbidities are common in long COVID patients. Certain immunomodulatory agents may impact glucose metabolism, potentially interfering with glycemic control in diabetic patients, while others could affect blood pressure regulation or lipid profiles, posing risks for cardiovascular patients (Alijotas-Reig et al., 2020; Bonilla et al., 2023). For example, corticosteroids, frequently used for inflammation, can exacerbate hyperglycemia and increase cardiovascular risks. On the other side, antidiabetic drug metformin may offer multiple therapeutic benefits for COVID-19, potentially improving treatment outcomes and reducing mortality. Beyond its primary role in lowering blood glucose and enhancing insulin sensitivity, metformin could inhibit viral entry, replication, and development, interfere with viral protein synthesis and host interactions, and modulate inflammation and immune responses in COVID-19 patients (Samuel et al., 2021). Understanding these interactions is essential to minimize adverse effects and optimize outcomes, necessitating tailored therapeutic strategies that account for the patient’s full medical profile.

Additionally, neuroprotective agents that can preserve neural function and prevent neurodegeneration may also be beneficial for COVID-19 survivors. These agents can include antioxidants, neurotrophic factors, and drugs that enhance synaptic plasticity. Clinical trials are needed to evaluate the efficacy of these agents in preventing or mitigating the long-term neurological effects of COVID-19.

Lifestyle interventions, such as regular physical activity, stress management, and a healthy diet, are crucial in mitigating the long-term effects of COVID-19 on neuroimmune aging. Exercise may have neuroprotective and anti-inflammatory effects, while a diet rich in antioxidants and anti-inflammatory compounds can boost immune and brain health (Di Benedetto et al., 2017; Shafqat et al., 2024). Additionally, modifying the gut microbiome through dietary interventions—like prebiotics (which promote beneficial bacterial growth), probiotics (live beneficial bacteria), synbiotics (a combination of prebiotics and probiotics), and postbiotics (fermentation byproducts)—can promote healthy aging (Conway and Duggal, 2021; Ji et al., 2023).

The advantages of mitochondrial rejuvenation as a gerotherapeutic approach are illustrated by the positive effects of exercise and caloric restriction. These interventions lead to increased mitochondrial biogenesis and oxidative capacity, resulting in enhanced immunometabolic health. In mice, these benefits are associated with increased longevity and an extended healthspan (Civitarese et al., 2007; Di Benedetto et al., 2017; Ross et al., 2019; Shafqat et al., 2024; Spadaro et al., 2022).

Stress management techniques such as mindfulness and meditation can reduce the psychological impact of the pandemic and support overall well-being. Combining meditation and yoga has been shown to normalize levels of the pro-inflammatory cytokine TNF-α (Epel et al., 2016). Meditation also increases concentrations of the anti-inflammatory cytokine IL-10 and decreases levels of pro-inflammatory IL-12. A meta-analysis revealed that mindfulness-based interventions positively affect cytokine levels related to low-grade inflammation (Bower et al., 2015; Cahn et al., 2017; Carlson et al., 2003; Jang et al., 2017) characterized aging. Studies investigating the effects of meditation on neurotransmitters and immune profiles have found various neuroimmune benefits (Sanada et al., 2020). Meditation may help patients by modulating pro-inflammatory to anti-inflammatory responses and decreasing the over-activation of the sympathetic nervous system, promoting relaxation (Carlson et al., 2003). Such methods stabilizing parasympathetic responses can be easily integrated into daily life to combat long COVID (Porter and Jason, 2022). The positive effects of regular exercise on neuroimmune heath include restored neurotransmission, remyelination, enhanced BBB integrity, and improved immune responses (Phillips et al., 2014; Razi et al., 2022). Improved BBB permeability is achieved through changes in tight junction proteins, increased activity of surrounding astrocytes, enhanced oxidative capacity of microglia, and reduced inflammation (Chupel et al., 2018; Souza et al., 2017). Additionally, exercise reduces autoimmunity, mitigates chronic inflammation, and suppresses microgliosis by elevating levels of anti-inflammatory cytokines (Brandt and Pedersen, 2010; Gleeson et al., 2011).

In conclusion, immunomodulatory therapeutic strategies, regular physical activity, stress management, and a healthy diet can lead to positive changes in the nervous and immune systems, creating an anti-inflammatory environment and promoting homeostasis, which may help mitigate the adverse effects of long COVID. While there is compelling evidence supporting the benefits of these interventions, optimal programs are still needed for individuals with persistent long COVID symptoms. These programs should include standardized treatment protocols, personalized care plans across various medical specialties, and controlled physical interventions to positively modulate disturbed neuroimmune responses, improve neuroimmune health and positively influence the post-COVID trajectory of aging.

## 5 Conclusion

The COVID-19 pandemic has underscored the complex relationship between viral infections, immune aging, and brain health. Long COVID is a multifaceted challenge that demands a coordinated, multidisciplinary approach to be effectively addressed. As we continue to grapple with the long-term impacts of COVID-19, it is crucial to deepen our understanding of how the virus may accelerate neuroimmune aging and develop interventions to mitigate these effects. Enhancing integration within healthcare systems, improving data collection, and conducting targeted research into the pathophysiology and treatment of long COVID are essential steps toward supporting affected individuals and minimizing the broader societal impact. Successfully addressing these challenges will be vital for improving the health outcomes of COVID-19 survivors and managing the broader implications for aging populations.
